# DNA Methylation-Associated Epigenetic Changes in Thermotolerance of *Bemisia tabaci* During Biological Invasions

**DOI:** 10.3390/ijms26157466

**Published:** 2025-08-01

**Authors:** Tianmei Dai, Yusheng Wang, Xiaona Shen, Zhichuang Lü, Fanghao Wan, Wanxue Liu

**Affiliations:** 1 Key Laboratory of Forest Bio-Resources and Integrated Pest Management for Higher Education in Hunan Province, College of Forestry, Central South University of Forestry and Technology, Changsha 410004, China; dai.tian.mei@163.com; 2 State Key Laboratory for Biology of Plant Diseases and Insect Pests, Key Laboratory for Prevention and Control of Invasive Alien Species of Ministry of Agriculture and Rural Affairs, Institute of Plant Protection, Chinese Academy of Agricultural Sciences, Beijing 100193, China; yushengwang01@163.com (Y.W.); naxiaoshen@163.com (X.S.); liuwanxue@caas.cn (W.L.); 3 Agricultural Genome Institute at Shenzhen, Chinese Academy of Agricultural Sciences, Shenzhen 518120, China

**Keywords:** DNA methylation, thermotolerance, rapid adaptation, DNA methyltransferases, biological invasion, *Bemisia tabaci*

## Abstract

Global warming and anthropogenic climate change are projected to expand the geographic distribution and population abundance of ectothermic species and exacerbate the biological invasion of exotic species. DNA methylation, as a reversible epigenetic modification, could provide a putative link between the phenotypic plasticity of invasive species and environmental temperature variations. We assessed and interpreted the epigenetic mechanisms of invasive and indigenous species’ differential tolerance to thermal stress through the invasive species *Bemisia tabaci* Mediterranean (MED) and the indigenous species *Bemisia tabaci* AsiaII3. We examine their thermal tolerance following exposure to heat and cold stress. We found that MED exhibits higher thermal resistance than AsiaII3 under heat stress. The fluorescence-labeled methylation-sensitive amplified polymorphism (F-MSAP) results proved that the increased thermal tolerance in MED is closely related to DNA methylation changes, other than genetic variation. Furthermore, the quantitative real-time polymerase chain reaction (qRT-PCR) and Western blotting analysis of DNA methyltransferases (Dnmts) suggested that increased expression of Dnmt3 regulates the higher thermal tolerance of female MED adults. A mechanism is revealed whereby DNA methylation enhances thermal tolerance in invasive species. Our results show that the Dnmt-mediated regulation mechanism is particularly significant for understanding invasive species’ successful invasion and rapid adaptation under global warming, providing new potential targets for controlling invasive species worldwide.

## 1. Introduction

Anthropogenic climate change has profound and diverse effects on ectotherm species globally, facilitating geographic expansion and increasing population size, exacerbating the effects of biological invasion [1,2,3]. It is becoming increasingly clear that temperature is a major driver of successful invasion [2]. The high physiological performance and rapid biological plasticity of invasive insects enable them to modify their phenotype in response to a wide range of environmental temperatures, leading to their successful establishment and spread in novel environments [4,5,6]. Additionally, invasive species with broader thermal tolerance have a greater ability to deal with thermal fluctuation compared to native species [7,8,9,10]. 

Plasticity responses to environmental temperature perturbations can be viewed at the epigenetic level as reversible, relatively short-term, and heritable changes in gene function [11,12]. Ectotherms, the bodies of which temperatures conform to ambient, differ widely in their temperature tolerance and ability to adjust to these limits. In ectothermic species, epigenetic mechanisms are known to affect gene expression regulation in response to temperature fluctuations during invasion. For instance, in pinewood nematodes (*Bursaphelenchus xylophilu*), changes in the abundance of 5-methylcytosine (5mC) and N6-methyladenine (6mA) showed the same trends in response to temperature change, but opposite trends during development. This indicates that DNA methylation plays crucial roles in the rapid adaptation of pinewood nematodes during invasion [13]. Importantly, invasive species nearly always demonstrate greater plasticity in order to adapt to areas with greater resource availability compared to non-invasive species, which are limited by their conditions [14,15]. Epigenetic modification responds more rapidly than genetic mutation, as such changes can occur in one generation. Thus, epigenetic changes may be the most significant determinants of environmentally induced phenotypic changes during successful invasions, rather than genetic variability [16]. DNA methylation allows ectotherms to cope with temperature stress on short time scales [17]. The rice leaf folder (*Cnaphalocrocis medinalis*) larvae can imprint their heat-stress memory through DNA methylation variation, leading to the transgenerational development of heat acclimation to higher temperatures [18]. DNA methylation commonly occurs at cytosine residues mediated by several conserved enzymes known as DNA methyltransferases (Dnmts). DNA methyltransferases Dnmt1 and Dnmt3 are the two main functional enzymes responsible for setting and maintaining DNA methylation patterns [19], while Dnmt2 is a tRNA methyltransferase [20,21,22]. The expression patterns of Dnmts can be altered by stress. For instance, in the tomato leaf miner (*Tuta absoluta*), the expression of *Dnmt1* was significantly altered by temperature stress treatments at different development stages [23]. Foundational work in zebrafish (*Danio rerio*) demonstrated that *Dnmt3a* and *Dnmt3b* may play different roles in the thermal epigenetic regulation of gene expression during early development [24]. Although the distribution of Dnmts differs across insect groups [25,26,27], Hemiptera contains a full DNA methylation tool kit, including all three Dnmt types [28,29]. Hence, DNA methylation may also play a critical role when ectotherms respond to thermal stimuli, including invasive insects.

The whitefly *Bemisia tabaci* is a globally destructive pest of agricultural crops. It comprises a species complex of multiple morphologically indistinguishable species [30,31,32]. In China, *B. tabaci* Mediterranean MED was first discovered in Yunnan in 2003 [33]. It successfully colonized and diffused to most provinces in China over the next 10 years, causing the widespread displacement of indigenous (*B. tabaci* AsiaII3) and invasive (*B. tabaci* Middle East-Asia Minor 1) species [34,35]. The successful invasion of MED is associated with its broader thermal tolerance [36,37,38,39], which is affected by several factors, including genetic variation (*heat shock proteins* and *transient receptor potential*) [40,41], endosymbiotic bacteria (*Rickettsia*) [42], plant viruses (tomato yellow leaf curl virus) [43], and microRNAs (Bta-miR-129) [44]. However, previous research on several invasive species suggests that epigenetic changes, rather than genetic variability, determine successful invasion [16,45,46]. The *B. tabaci* genome encodes a full DNA methylation tool kit, including all three methyltransferases [47,48]. Thus, we hypothesize that the higher thermal tolerance and invasiveness of MED are related to epigenetic variation. 

In the present study, we examined the thermal tolerance of the invasive species MED and indigenous species AsiaII3 when exposed to heat and cold stress for one generation. After thermal exposure, we screened the epigenetic and genetic variations evident in both MED and AsiaII3 with fluorescence-labeled methylation-sensitive amplified polymorphism (F-MSAP). We elucidated the possible molecular mechanism driving the epigenetic differences by examining the Dnmt mRNA and protein expression levels of both MED and AsiaII3 after thermal exposure. Our findings allow expanded scientific understanding of the epigenetic mechanisms that may underlie the thermal-induced phenotypic plasticity observed in MED and better explain and predict its rapid adaptation and expansion, with implications for the spread and management of other invasive ectotherms.

## 2. Results

### 2.1. Responses to Thermal Exposure

**Figure 1:**
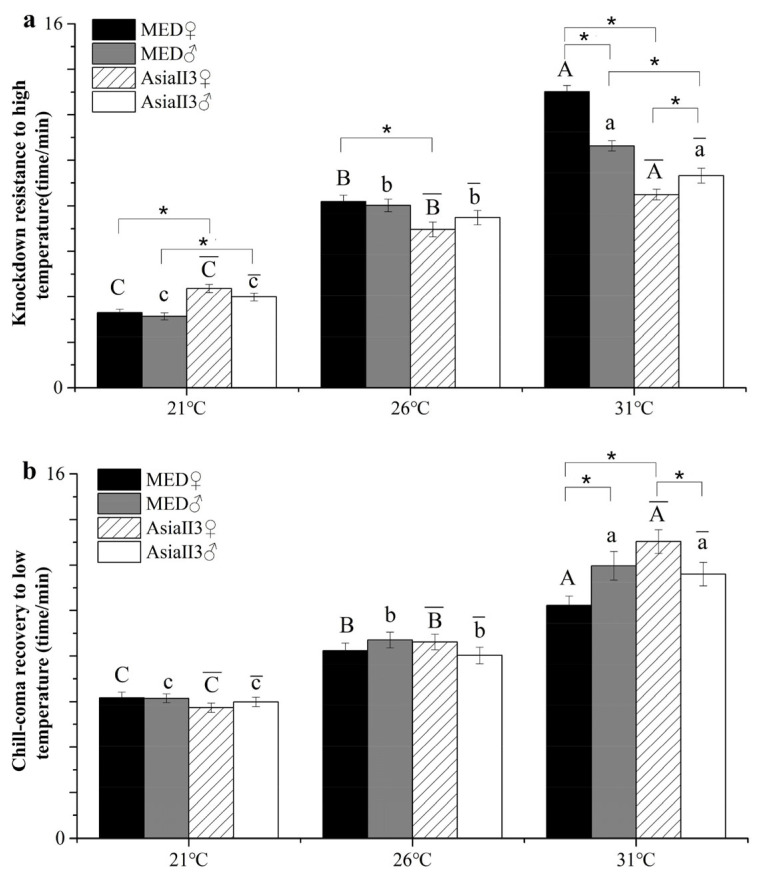
Heat knockdown time and chill-coma recovery time after thermal exposure for one generation of MED and AsiaII3. (**a**) The time of knockdown resistance to high temperature for females and males of MED and AsiaII3; (**b**) the time of chill-coma recovery to low temperature in females and males of MED and AsiaII3. Data are presented as the means ± standard error of 100 replicates. Uppercase letters atop the bars indicate a significant difference in MED females among different temperature treatments; lowercase letters atop the bars indicate the significant difference in MED males among different temperature treatments; uppercase letters with overlines atop the bars indicate a significant difference in AsiaII3 females among different temperature treatments; lowercase letters with overlines atop the bars indicate a significant difference in AsiaII3 males among different temperature treatments (ANOVA, *p* < 0.05). * indicates a statistically significant difference (Student’s *t*-test, *p* < 0.05).

There was a significant interaction effect of species × gender × temperature in knockdown resistance to high temperature (KRHT) and chill-coma recovery to low temperature (CCRT) (F_KRHT_ = 12.63, *p* < 0.05; F_CCRT_ = 7.731, *p* < 0.05). All individuals responded to thermal stress (Figure 1), with significantly higher KRHT and CCRT compared to those raised at 31 °C and significantly lower knockdown resistance for the 21 °C treatment than the control treatment (26 °C). Sex played a key role at higher temperatures in both KRHT and CCRT. In addition, MED females raised at 31 °C exhibited higher heat resistance than males, while the opposite was true of AsiaII3 (Figure 1a). We also compared MED and AsiaII3 of each sex. KRHT was more variable in MED compared to AsiaII3; it was significantly higher for females raised at 26 °C or 31 °C and for males raised at 31 °C, but lower in both females and males raised at 21 °C (Figure 1a). CCRT in MED was higher in females raised at 31 °C than in the same group of AsiaII3 (Figure 1b).

### 2.2. Epigenetic Variation Between MED and AsiaII3 After Thermal Exposure

A total of 3456 F-MSAP sites resolved by 16 primer combinations were detected by F-MSAP in MED and AsiaII3 females and males after heat and cold stress (Table 1, Table S1). We detected hemi-methylation (type II) ranging from 6.19% to 12.30%, and full methylation (type III) ranging from 17.16% to 33.33% (Table 1). In general, the hemi-methylation ratio in MED males increased considerably after cold (21 °C) and heat (31 °C) stress, whereas it decreased in MED females. The hemi-methylation ratio also increased at 21 °C and 31 °C in AsiaII3 males and at 21 °C in females, but decreased at 31 °C in females.

Epigenetic and genetic variations revealed the sex and species patterns of DNA methylation/demethylation differences (Table 2, Figures S1 and S2). No global epigenetic differences (MSL) were detected in MED. For AsiaII3, only the control group (26 °C) differed significantly (Φ_ST_ = 0.095, *p* = 0.001). Furthermore, MSL showed significant differences between MED and AsiaII3 females raised at 21 °C (Φ_ST_ = 0.063, *p* = 0.020, AMOVA) and 31 °C (Φ_ST_ = 0.093, *p* = 0.002) and between males raised at 31 °C (Φ_ST_ = 0.050, *p* = 0.022) (Table 2, Figures S1 and S2). Genetic variation (NML) differed only for the MED control group (26 °C) (Φ_ST_ = 0.099, *p* < 0.001). NML between MED and AsiaII3 showed significant differences for both females (Φ_ST_ = 0.019, *p* = 0.023) and males (Φ_ST_ = 0.019, *p* = 0.041) at 31 °C (Table 2, Figures S3 and S4). Epigenetic diversity (MSL) was significantly higher than genetic diversity (NML), with Shannon’s index of MSL appearing higher than NML for each comparison (*p* < 0.001) (Table 2).

### 2.3. Expression of Dnmts Across Tagmata, Development, and Sex

**Figure 2:**
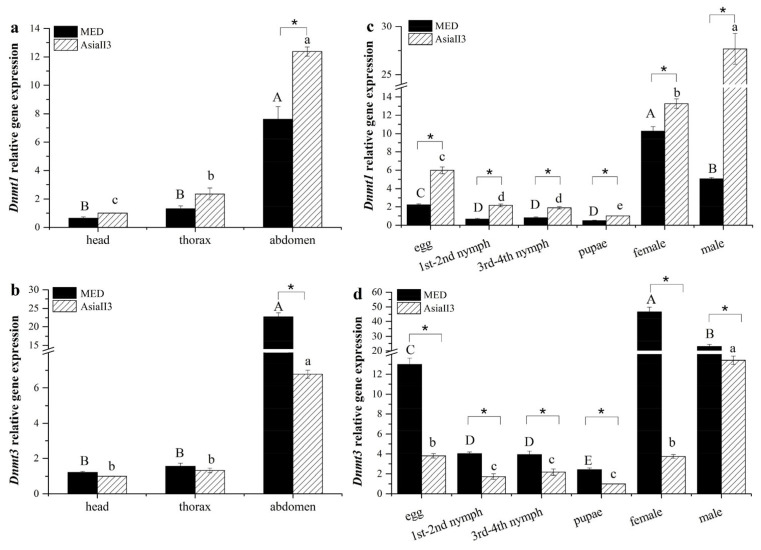
The tagmata and developmental expression of *Dnmt1* and *Dnmt3* in MED and AsiaII3. (**a**) The tagmata expression of *Dnmt1* in MED and AsiaII3; (**b**) the tagmata expression of *Dnmt3* in MED and AsiaII3; (**c**) the developmental expression of *Dnmt1* in MED and AsiaII3; (**d**) the developmental expression of *Dnmt3* in MED and AsiaII3. Uppercase letters atop the bars indicate a significant difference in MED among different tagmata or developmental stages; lowercase letters atop the bars indicate a significant difference in AsiaII3 among different tagmata or developmental stages (ANOVA, *p* < 0.05). The results are expressed as the mean ± the SEM. * indicates significant differences between MED and AsiaII3 (Student’s *t*-test, *p* < 0.05). The head and pupae of AsiaII3 were used as calibrator samples for the tagmata or developmental expression, respectively.

The transcripts of both *Dnmt1* and *Dnmt3* were expressed abundantly in the abdomen and at relatively low levels in the head and thorax in both species. *Dnmt1* was expressed twice as much in the abdomen in AsiaII3 than in MED (Figure 2a). In contrast, *Dnmt3* showed a three-fold increase in the abdomen in MED compared to AsiaII3 (Figure 2b).

Both *Dnmt1* and *Dnmt3* were expressed at significantly higher levels in the adult stage than in any other stage in each species. The expression of *Dnmt1* was significantly higher in the egg and adult stages of AsiaII3 than in those of MED. Conversely, the expression of *Dnmt3* was significantly lower in the egg and adult stages of AsiaII3 than in those of MED. There were dramatic differences in *Dnmt* expression across both sexes and species: both *Dnmt1* and *Dnmt3* were expressed twice as much in the males as the females of AsiaII3, while the opposite pattern was observed in MED (Figure 2c,d).

### 2.4. Expression of Dnmts in MED and AsiaII3 After Thermal Exposure

**Figure 3:**
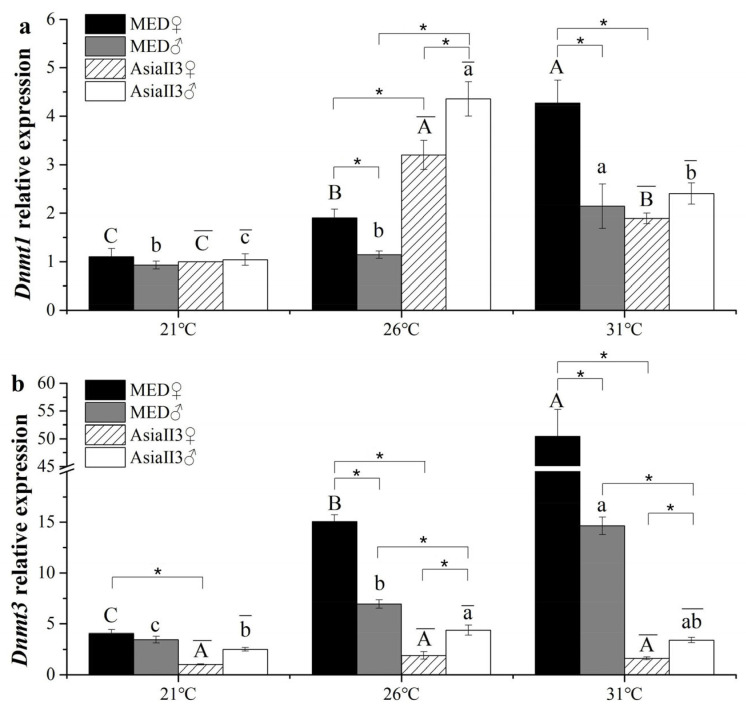
The relative expression levels of Dnmt1 and Dnmt3 in MED and AsiaII3 after thermal exposure for one generation. (**a**) The relative expression of Dnmt1 in females and males in MED and AsiaII3; (**b**) the relative expression of Dnmt3 in females and males in MED and AsiaII3. Uppercase letters atop the bars indicate a significant difference in MED females among different temperature treatments; lowercase letters atop the bars indicate a significant difference in MED males among different temperature treatments; uppercase letters with overlines atop the bars indicate a significant difference in AsiaII3 females among different temperature treatments; lowercase letters with overlines atop the bars indicate a significant difference in AsiaII3 males among different temperature treatments (ANOVA, *p* < 0.05). The results are expressed as the mean ± the SEM. * indicates a significant difference (Student’s *t*-test, *p* < 0.05). AsiaII3 females exposed to 21 °C were used as the calibrator sample.

In MED, *Dnmt1* and *Dnmt3* mRNA expression levels were significantly down-regulated after cold stress, but up-regulated after heat stress (Figure 3). However, in AsiaII3, *Dnmt1* expression levels were significantly down-regulated after heat and cold stress (Figure 3a), while *Dnmt3* expression was only down-regulated in females raised at 21 °C (Figure 3b). *Dnmt3* expression levels differed significantly between females and males at each temperature in both MED and AsiaII3. For MED, *Dnmt1* was expressed twice as much in females as in males raised at 31 °C, and *Dnmt3* was over three-fold more highly expressed in females than in males raised at 31 °C. The expression level of *Dnmt1* in MED was significantly higher than that in AsiaII3 in females raised at 31 °C (Figure 3a). The expression level of *Dnmt3* in MED was significantly higher than that in AsiaII3 females raised at 21 °C and 31 °C and in males raised at 31 °C (Figure 3b).

### 2.5. Expression of Dnmt Proteins in MED and AsiaII3 After Thermal Exposure

**Figure 4:**
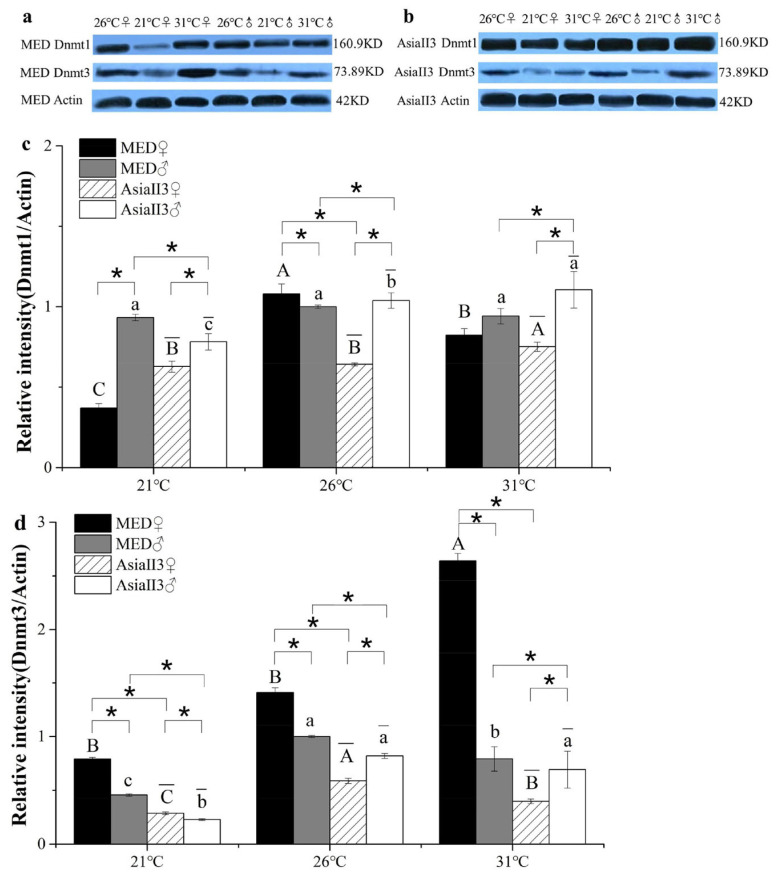
Western blot analyses of Dnmt1 and Dnmt3 in MED and AsiaII3 after thermal exposure for one generation. (**a**) Representative blots in MED; (**b**) representative blots in AsiaII3; (**c**) the quantification of the band intensity of Dnmt1 in females and males of MED and AsiaII3; (**d**) the quantification of band intensity of Dnmt3 in females and males of MED and AsiaII3. Uppercase letters atop the bars indicate a significant difference in MED females among different temperature treatments; lowercase letters atop the bars indicate a significant difference in MED males among different temperature treatments; uppercase letters with overlines atop the bars indicate a significant difference in AsiaII3 females among different temperature treatments; lowercase letters with overlines atop the bars indicate a significant difference in AsiaII3 males among different temperature treatments (ANOVA, *p* < 0.05). The results are expressed as the mean ± the SEM. * indicates a significant difference (Student’s *t*-test, *p* < 0.05). MED males exposed to 26 °C were used as the calibrator sample.

The expression of Dnmt1 and Dnmt3 proteins was analyzed using Western blotting (Figure 4). The SDS-PAGE autoradiogram of immunoprecipitated Dnmt1 and Dnmt3 proteins revealed major ∼160 kD and 74 kD bands, respectively (Figure 4a,b). Dnmt1 was significantly decreased in MED females after heat and cold stress but showed no differences within MED males (Figure 4c). By contrast, Dnmt1 expression increased in AsiaII3 females and males raised at 31 °C but decreased in males raised at 21 °C (Figure 4c). In addition, Dnmt3 expression significantly increased in MED females at 31 °C and decreased in MED males raised at 21 °C and 31 °C (Figure 4d). Dnmt3 expression in AsiaII3 decreased in females and males raised at 21 °C and in females raised at 31 °C (Figure 4d). Furthermore, the expression of Dnmt1 and Dnmt3 was almost always significantly different between females and males at each temperature in both MED and AsiaII3 (Figure 4c,d). Dnmt3 expression was significantly higher in MED females raised at 31 °C than in AsiaII3 (Figure 4c,d).

## 3. Discussion

Climate change has profound effects on ectothermic species; increased temperatures are a major driver of invasion success [2,18]. Thus, understanding differential responses to temperature variation is critical to predicting how invasive species rapidly adapt through phenotypic plasticity and replace indigenous species [49,50]. Here, we chose the invasive species MED and the indigenous species AsiaII3 to examine thermal tolerance, epigenetic variations following exposure, and Dnmt mRNA and protein expression levels after heat and cold stress.

The invasive MED was more tolerant of high temperatures than the native species AsiaII3. Heat stress increased heat resistance but decreased cold resistance for both species. The higher temperature tolerance of MED is consistent with the findings of previous studies in this species complex [36,39]. Our findings have important implications for understanding the distribution and abundance of these two whitefly species. MED originates in southern Spain and Portugal and occurs primarily in tropical and subtropical zones [51,52,53], indicating that MED is a heat-resistant species. This proved to be an advantage after MED invaded China, as it rapidly spread to most provinces, except Jilin [35,54]. AsiaII3 is an indigenous species primarily distributed in the east and central south of China [35], which experiences a smaller range of temperature fluctuation. Furthermore, Flores et al. [55] observed that continuous short-term exposure to a consistent environment leads parent species to transmit beneficial survival strategies and phenotypes to their offspring. It is also possible that the short-term adaptive responses of invasive species to environmental changes are associated with epigenetics [45,56,57]. Thus, the rapid alteration in MED thermotolerance is likely influenced by epigenetic regulation. We found that MED females exhibit higher heat resistance than other groups. MED showed increased tolerance at high temperatures, which might be caused by changes in females. Similar sex differences are consistently found in other insects [58,59,60]. For example, female mosquitoes (*Aedes aegypti*) have a higher tolerance to short-term thermal stress than males [61]. The difference between the sexes of the two whitefly species in response to high temperatures may be a result of a difference in molecular mechanisms, which requires further study.

Epigenetic processes, such as DNA methylation, are highly dynamic in response to environmental stimuli and developmental changes, especially over ecological timescales [11,62,63]. The phenotypic results in this study confirm that MED and AsiaII3 have strikingly different responses to thermal stress, especially in females. Corresponding differences in DNA methylation between MED and AsiaII3 after thermal stress were detected using F-MSAP. AMOVA also revealed significant global epigenetic differences between MED and AsiaII3 species raised at 31 °C. Epigenetic diversity (MSL) was significantly higher than genetic diversity (NML) in both species. This pattern has been observed in other invasive species, including Japanese knotweed (*Fallopia japonica*) [64], house sparrows (*Passer domesticus*) [65,66], the Asian tiger mosquito (*Aedes albopictus*) [11], and the Australian tubeworm (*Ficopomatus enigmaticus*) [67]. Therefore, DNA methylation is an important source of phenotypic variation for adaptation to environmental conditions, particularly for the short timescales in which invasions occur. Phenotypic differences in female MED and AsiaII3 species were reflected in DNA methylation, which may be a key driver of increased phenotypic plasticity in response to thermal stress. These changes are then transmitted to offspring via the maternal line, enhancing their ability to cope with temperature stress [55,68,69].

The methylation of cytosines is catalyzed by the DNA methyltransferases Dnmt1 and Dnmt3. *Dnmt1* and *Dnmt3* were expressed more highly in the abdomen and adult stage of both MED and AsiaII3. Both genes are expressed in the posterior region, are related to reproduction [48,70,71], and play an important role in early development. *Dnmt1* expression was significantly higher in AsiaII3 than MED adults and was more highly expressed in males compared to females in AsiaII3. Interestingly, the expression of Dnmt1 is entirely the opposite in MED. As *Dnmt1* is consistently related to reproduction in other species [72,73], the high expression of *Dnmt1* in female MED indicates its involvement not only in fundamental oocyte development [74] but also in modulating MED’s temperature tolerance [47]. Nevertheless, the divergence between the Dnmt1 protein and mRNA expression levels likely underscores the complexity of Dnmt1 expression regulation in MED, suggesting the potential existence of multi-tiered gene expression control that requires further exploration.

*Dnmt3* expression was significantly low in AsiaII3 adults overall, though it was more highly expressed in male AsiaII3 and female MED. In invasive MED, *Dnmt3* expression was increased, especially in females. The expression patterns of Dnmt mRNA and protein differed after thermal stress in MED and AsiaII3. Dnmt1 functions as a maintenance methyltransferase, which is less important than Dnmt3 in the invasive species MED. The de novo methyltransferase Dnmt3 plays a critical role in the regulation of phenotypic plasticity [75,76,77]. Hence, Dnmt3 may confer ecological advantages to the invasive species MED, which has the potential to optimize plasticity and acclimation in response to different environments. Variants of *Dnmt3* optimize plasticity across sex and development in response to environmental variation, such as in the brown planthopper (*Nilaparvata lugens*) [29] and in zebrafish [77]. Our previous study demonstrated that both heat and cold resistance significantly decrease after the knockdown of *Dnmt3* in *B. tabaci* [48]. We, therefore, speculate that the *Dnmt3* gene is crucial in the response of MED to high-temperature environments, which enables female adults to acclimatize to thermal stress and transfer this resistance to offspring via de novo Dnmt3.

## 4. Materials and Methods

### 4.1. Insect Materials

Two whitefly species, *B. tabaci* MED and AsiaII3, were collected in July 2012 from Beijing and Zhejiang, China, respectively. Insects were maintained on cotton (*Gossypium hirsutum* (Chuangyou 168)) in cages in an insectary at 26 ± 2 °C with 50 ± 10% relative humidity (RH) and a 14 L:10 D photoperiod. Both species were maintained in the laboratory for 20–30 generations when used in the experiments.

### 4.2. Thermal Stress

Approximately 1000 newly emerged adults were released into cages with fresh cotton plants and allowed to oviposit for 72 h at 26 °C in climate-controlled chambers (Saifu, Ningbo, China). Adults were then removed, and the newly laid eggs were exposed to low (21 °C), high (31 °C), or control (26 °C) temperatures from development to the adult stage. All temperature regimes were set with 50 ± 10% relative humidity and a photoperiod of 14 L:10 D. After thermal exposure, half of the newly emerged adults were frozen immediately in liquid nitrogen for RNA and DNA extraction, while the other half were used for thermal tolerance analysis.

### 4.3. Tagmata and Developmental Sample Collection

Expression levels were evaluated across the tagmata and developmental stages to understand the temporal and spatial characteristics of Dnmt gene expression in MED and AsiaII3. For the three major tagmata samples, the head, thorax, and abdomen were separately collected from 400 female and 400 male adults. For the five different developmental stages, 2000 eggs, 1000 1st–2nd nymphs, 500 3rd–4th nymphs, 500 pupae, and 200 female and 200 male adults were collected from plants. All samples were frozen immediately in liquid nitrogen and stored at −80 °C until RT-qPCR was used to evaluate the expression of their mRNA transcripts.

### 4.4. Thermal Tolerance Analysis

To compare the heat and cold resistance between species and sexes, newly emerged individuals were randomly selected and scored for thermal resistance. Knockdown resistance to high temperature (KRHT) and chill-coma recovery to low temperature (CCRT) are commonly used measures to assess thermal resistance in insects and other organisms [38,78,79]. One adult whitefly was confined in a 5 mL centrifuge tube capped with a cotton pad. KRHT was measured as knockdown time at 45 °C (the time at which the whitefly lost control of its body and fell to the bottom of the tube), while CCRT was measured as the time to recovery from cold treatment for 10 min at −5 °C [38]. To avoid possible circadian effects, all whiteflies used had emerged for 72 h, and tests were performed between 10 a.m. and 2 p.m.

### 4.5. DNA Extraction and F-MSAP Analysis

The improved salting-out method was used to extract whitefly genome DNA [80]. F-MSAP analysis was adopted to detect epigenetic markers, in accordance with the methods of Xiong et al. [81] and Yang et al. [82]. The restriction endonuclease combinations EcoRI/HpaII and EcoRI/MspI were used for double digestion, and the 16 fluorescently labeled primer pairs used for pre-amplification and selective amplification are summarized in Supplemental Table S1. Methylation-sensitive polymorphic fragments were generated and detected using 4% denaturing PAGE run on an ABI PRIS M377 DNA sequencer (Applied Biosystems, Foster, CA, USA). Finally, all bands generated from GeneScan 3.1 software were transformed into a binary data matrix according to presence (1) or absence (0). Four patterns of methylation were defined according to the following emerging bands: Type I—non-methylation (E-H/E-M, 1/1); Type II—hemi-methylation (E-H/E-M, 1/0); Type III—full methylation (E-H/E-M, 0/1); and Type IV—uninformative methylation (E-H/E-M, 0/0) [83]. These were then classified into methylation-susceptible loci (MSL) and non-methylated loci (NML), which were analyzed to assess epigenetic variation and genetic variation, respectively.

### 4.6. Total RNA Isolation and Quantitative Real-Time PCR

Total RNA was isolated using the TRIzol reagent (Invitrogen, Carlsbad, CA, USA), and the concentration and quality of RNA were examined using NanoPhotometer^TM^ P330 (Implen, Munich, Germany). RNA was then reverse transcribed using the Super Script First-Strand Synthesis System (Transgen Biotech, Beijing, China) and stored at −20 °C. *β-Tubulin* was used as the reference gene along with established primers [47,48].

### 4.7. Western Blot Analysis

The total protein of each adult was extracted using a Tissue or Cell Total Protein Extraction Kit (Sangon Biotech, Shanghai, China), and the protein concentration was determined using a BCA Protein Assay Kit (Sangon Biotech) according to the instructions. Samples (40 μg) were separated by an SDS polyacrylamide gel electrophoresis (SDS-PAGE) gel, blotted on polyvinylidene difluoride membranes (Ewell, Guangzhou, China), and hybridized using the following antibodies: anti-Dnmt1 at 1:1250 dilution, anti-Dnmt3 at 1:1250 dilution, and anti-Actin at 1:1000 dilution. Then the membranes were incubated with HRP-conjugated Goat Anti-Rabbit IgG (Sangon Biotech) and IgG (H + L) HRP (ERWAN, Shanghai, China) secondary antibodies (1:8000). The immunoreactive bands were visualized using an ECL detection kit (Sangon Biotech). Band intensities were quantified using densitometry (Quantity One version 4.6.7; Bio-Rad, Hercules, CA, USA).

### 4.8. Statistical Analysis

Analyses were conducted with the SPSS v. 16.0 software package (SPSS Inc., Chicago, IL, USA). The knockdown and recovery data were first tested for normality using the Kolmogorov–Smirnov test and were log-transformed to ensure a normal distribution. Comparison of the thermal tolerance of species was performed using a one-way analysis of variance (ANOVA), followed by the Tukey–Kramer multiple comparison test. Comparisons of the differences in thermal tolerance across species and sexes were analyzed using an independent-samples Student’s *t*-test. F-MSAP individual and species profiles were analyzed using the R package *msap* v.3.2.2.39 [84]. Epigenetic (MSL) and genetic (NML) variation were estimated using the Shannon diversity index (I) and the differentiation phi coefficient (Φ_ST_). The expression of the target gene mRNAs and proteins was analyzed using one-way ANOVA, followed by the Tukey–Kramer test and independent-samples Student’s *t*-test. Significance was defined at *p* < 0.05. Data are presented as the means ± standard error of three replicates.

## 5. Conclusions

Our study showed that the invasive MED has a higher thermal tolerance than the indigenous AsiaII3. Importantly, DNA methylation variation, especially Dnmt3 expression, is responsible for differences in thermal tolerance between the two whitefly species. In invasive MED species, the epigenetic control system is a major driver of increased thermotolerance; stress-induced Dnmt variation enables this species to adapt rapidly to environmental temperature during biological invasions. These findings enhance our understanding of the epigenetic temperature adaptation common to many invasive ectotherm species and will aid in better predicting and limiting the climate change-facilitated spread of invasive insect species. Further research should examine whether DNA methylation and changes in Dnmt expression are drivers of the range expansion of similarly pernicious agricultural pests in other systems.

## Figures and Tables

**Table 1:** DNA methylation patterns in MED and AsiaII3 after thermal exposure for one generation.

Temperature Treatment	Species and Gender	Types				Hemi-Methylation Ratio ^1^	Full Methylation Ratio ^2^	Total Methylation Ratio ^3^
		I (1/1)	II (1/0)	III (0/1)	IV (0/0)			
21 °C	MED♀	771	291	307	2087	21.26%	22.43%	43.69%
	MED♂	779	346	233	2098	25.48%	17.16%	42.64%
26 °C	MED♀	628	358	450	2020	24.95%	31.33%	56.28%
	MED♂	684	214	325	2233	17.49%	26.59%	44.08%
31 °C	MED♀	669	266	244	2277	22.56%	20.70%	43.26%
	MED♂	634	425	284	2113	31.65%	21.15%	52.80%
21 °C	AsiaII3♀	516	322	324	2294	27.70%	27.87%	55.57%
	AsiaII3♂	689	416	259	2091	30.51%	18.99%	49.50%
26 °C	AsiaII3♀	584	304	356	2212	24.44%	28.62%	53.06%
	AsiaII3♂	606	278	394	2178	21.75%	30.83%	52.58%
31 °C	AsiaII3♀	821	237	251	2146	18.13%	19.17%	37.30%
	AsiaII3♂	603	377	440	2036	26.57%	30.97%	57.54%

^1^ Hemi-methylation ratio = II/(I + II + III); ^2^ full methylation ratio = III/(I + II + III); ^3^ total methylation ratio = (II + III)/(I + II + III).

**Table 2:** Epigenetic and genetic differentiation in MED and AsiaII3 after thermal exposure for one generation.

Temperature Treatment	Species and Gender	Shannon’s Diversity Index		Φ_ST_ ^4^		W ^3^
		MSL ^1^	NML ^2^	MSL ^1^	NML ^2^	
21 °C	MED♀ vs. MED♂	0.643 ± 0.056	0.450 ± 0.161	−0.015 (*p* = 0.722)	−0.044 (*p* = 1.000)	9013 (*p* < 0.001)
26 °C	MED♀ vs. MED♂	0.663 ± 0.040	0.365 ± 0.134	0.023 (*p* = 0.170)	0.099 (*p* < 0.001)	10,850 (*p* < 0.001)
31 °C	MED♀ vs. MED♂	0.631 ± 0.076	0.439 ± 0.164	0.017 (*p* = 0.234)	−0.040 (*p* = 1.000)	8842 (*p* < 0.001)
21 °C	AsiaII3♀ vs. AsiaII3♂	0.654 ± 0.058	0.385 ± 0.158	0.001 (*p* = 0.455)	−0.041 (*p* = 1.000)	10,073 (*p* < 0.001)
26 °C	AsiaII3♀ vs. AsiaII3♂	0.646 ± 0.059	0.373 ± 0.140	0.096 (*p* = 0.001)	0.015 (*p* = 0.078)	10,470 (*p* <0.001)
31 °C	AsiaII3♀ vs. AsiaII3♂	0.643 ± 0.067	0.371 ± 0.155	0.015 (*p* = 0.269)	−0.045 (*p* = 1.000)	9372 (*p* < 0.001)
21 °C	MED♀ vs. AsiaII3♀	0.653 ± 0.060	0.437 ± 0.160	0.005 (*p* = 0.3903)	−0.004 (*p* = 0.645)	10,078 (*p* < 0.001)
26 °C	MED♀ vs. AsiaII3♀	0.647 ± 0.056	0.394 ± 0.141	0.040 (*p* = 0.064)	0.027 (*p* = 0.007)	10,519 (*p* < 0.001)
31 °C	MED♀ vs. AsiaII3♀	0.644 ± 0.072	0.413 ± 0.160	0.093 (*p* = 0.002)	0.019 (*p* = 0.023)	10,337 (*p* < 0.001)
21 °C	MED♂ vs. AsiaII3♂	0.644 ± 0.063	0.426 ± 0.153	0.063 (*p* = 0.020)	0.014 (*p* = 0.079)	10,147 (*p* < 0.001)
26 °C	MED♂ vs. AsiaII3♂	0.657 ± 0.046	0.338 ± 0.141	0.105 (*p* < 0.001)	0.089 (*p* < 0.001)	10,059 (*p* < 0.001)
31 °C	MED♂ vs. AsiaII3♂	0.655 ± 0.053	0.409 ± 0.145	0.050 (*p* = 0.022)	0.019 (*p* = 0.041)	10,533 (*p* < 0.001)

^1^ MSL: methylation-susceptible loci; ^2^ NML: non-methylated loci; ^3^ W: Wilcoxon rank-sum test with continuity correction; ^4^ Φ_ST_: differentiation phi coefficient statistics.

## Data Availability

All data are contained within the article.

## Supplementary Materials

The supporting information can be downloaded at https://www.mdpi.com/article/10.3390/ijms26157466/s1.
