# Supplementary material for: DNA Methylation-Associated Epigenetic Changes in Thermotolerance of Bemisia tabaci During Biological Invasions
Source: Int J Mol Sci. 2025 Aug 1;26(15):7466. doi: 10.3390/ijms26157466 (PMC12347698; doi:10.3390/ijms26157466)
Supplement: Supplementary file 1 [file ijms-26-07466-s001.zip › ijms-3734241-supplementary.pdf]

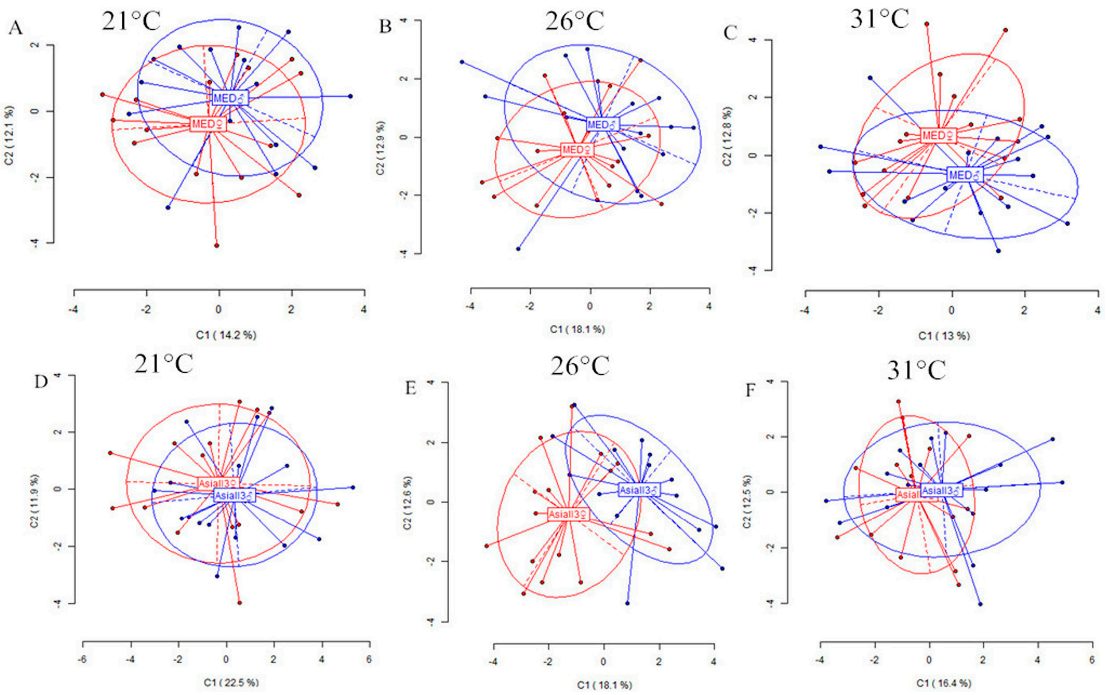

Supplementary Figure S1 PCA of females and males of MED and AsiaII3 after thermal exposure for one generation with epigenetic variation (methylation-sensitive loci). (A) Comparison of females and males MED exposed to 21 °C; (B) Comparison of females and males MED exposed to 26 °C; (C) Comparison of females and males MED exposed to 31 °C; (D) Comparison of females and males AsiaII3 exposed to 21 °C; (E) Comparison of females and males AsiaII3 exposed to 26 °C; (F) Comparison of females and males AsiaII3 exposed to 31 °C. Red symbols represent female individuals; blue symbols represent male individuals. Ellipses indicate the dispersion of these symbols around their centers. The long and short axes show the direction of maximum dispersion and minimum dispersion, respectively.

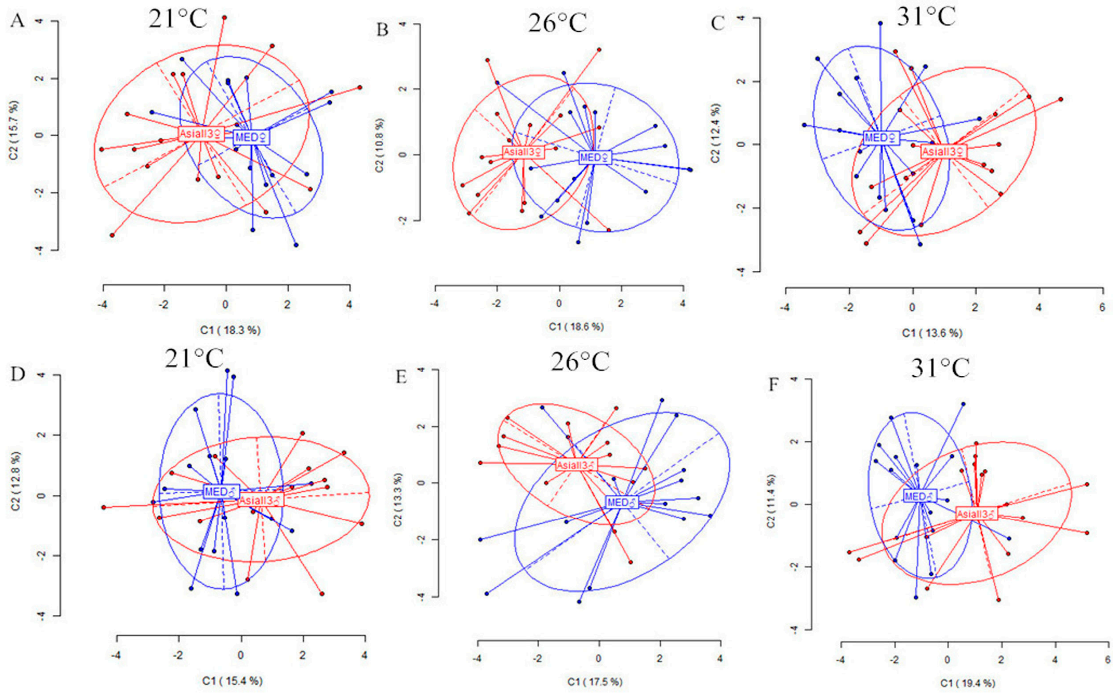

Supplementary Figure S2 PCA of MED and AsiaII3 after thermal exposure for one generation with epigenetic variation (methylation-sensitive loci). (A) Comparison of MED and AsiaII3 females exposed to 21 °C; (B) Comparison of MED and AsiaII3 females exposed to 26 °C; (C) Comparison of MED and AsiaII3 females exposed to 31 °C; (D) Comparison of MED and AsiaII3 males exposed to 21 °C; (E) Comparison of MED and AsiaII3 males exposed to 26 °C; (F) Comparison of MED and AsiaII3 males exposed to 31 °C. Red symbols represent AsiaII3 individuals; blue symbols represent MED individuals. Ellipses indicate the dispersion of these symbols around their centers. The long and short axes show the direction of maximum dispersion and minimum dispersion, respectively.

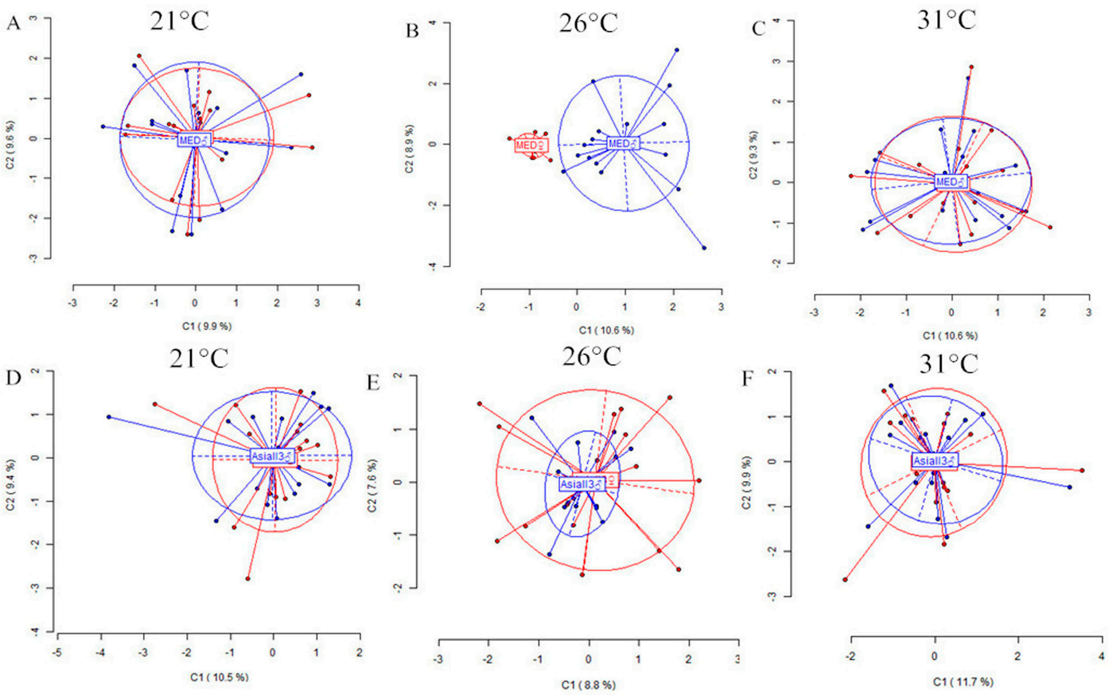

Supplementary Figure S3 PCA of females and males in MED and AsiaII3 after thermal exposure for one generation with genetic variation (non-methylation loci). (A) Comparison of females and males MED exposed to 21 °C; (B) Comparison of females and males MED exposed to 26 °C; (C) Comparison of females and males MED exposed to 31 °C; (D) Comparison of females and males AsiaII3 exposed to 21 °C; (E) Comparison of females and males AsiaII3 exposed to 26 °C; (F) Comparison of females and males AsiaII3 exposed to 31 °C. Red symbols represent female individuals; blue symbols represent male individuals. Ellipses indicate the dispersion of these symbols around their centers. The long and short axes show the direction of maximum dispersion and minimum dispersion, respectively.

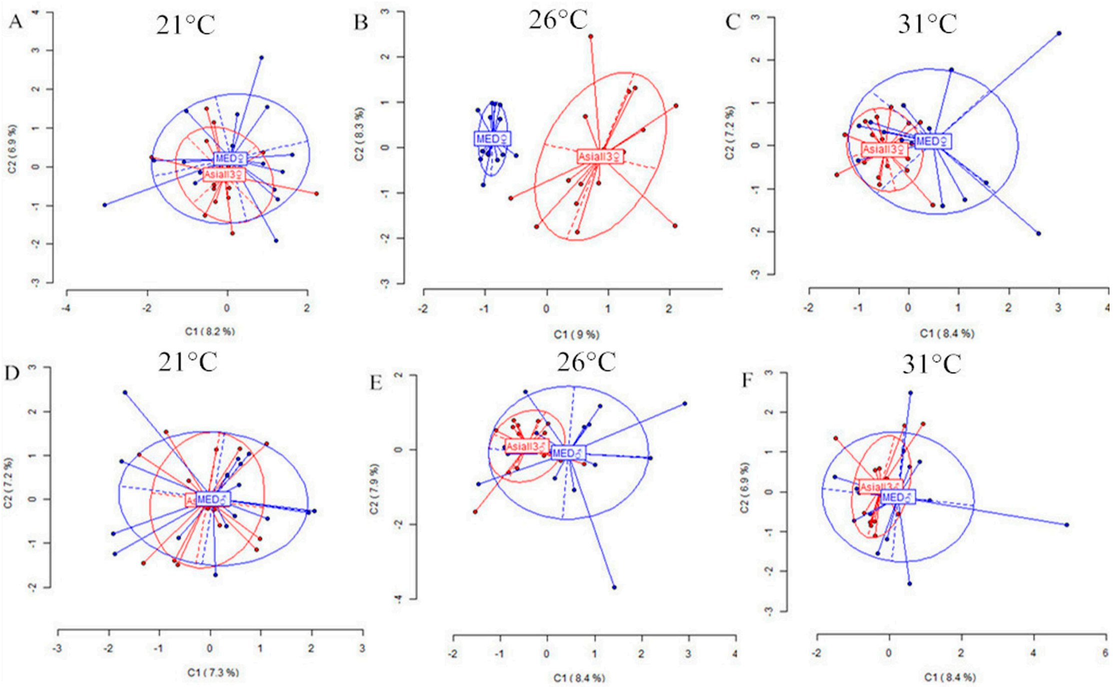

Supplementary Figure S4 PCA of MED and AsiaII3 after thermal exposure for one generation with the genetic variation (non-methylation loci). (A) Comparison of MED and AsiaII3 females exposed to 21°C; (B) Comparison of MED and AsiaII3 females exposed to 26 °C; (C) Comparison of MED and AsiaII3 females exposed to 31 °C; (D) Comparison of MED and AsiaII3 males exposed to 21°C; (E) Comparison of MED and AsiaII3 males exposed to 26 °C; (F) Comparison of MED and AsiaII3 males exposed to 31 °C. Red symbols represent AsiaII3 individuals; blue symbols represent MED individuals. Ellipses indicate the dispersion of these symbols around their centers. The long and short axes show the direction of maximum dispersion and minimum dispersion, respectively.

Supplementary Table S1 Adaptor and primer sequences used in F-MASP

**Sequence name**	***EcoR* I(E)**	***Hpa* II/*Msp* I(H/M)**
Adaptor-1	CTCGTAGACTGCGTACC	GATCATGAGTCCTGCT
Adaptor-2	AATTGGTACGCAGTC	CGAGCAGGACTCATGA
Pre-amplificaion primers	GACTGCGTACCAATTCA	ATCATGAGTCCTGCTCGG
Selective-amplification primers		
1st primer combination	FAM[2]-GACTGCGTACCAATTCAAC	ATCATGAGTCCTGCTCGGTCG
2nd primer combination	FAM[2]-GACTGCGTACCAATTCAAC	ATCATGAGTCCTGCTCGGTTA
3rd primer combination	FAM[2]-GACTGCGTACCAATTCAAC	ATCATGAGTCCTGCTCGGTGA
4th primer combination	FAM[2]-GACTGCGTACCAATTCAAC	ATCATGAGTCCTGCTCGGTAC
5th primer combination	FAM[2]-GACTGCGTACCAATTCAAG	ATCATGAGTCCTGCTCGGTGT
6th primer combination	FAM[2]-GACTGCGTACCAATTCACA	ATCATGAGTCCTGCTCGGTGA
7th primer combination	FAM[2]-GACTGCGTACCAATTCACA	ATCATGAGTCCTGCTCGGTGT
8th primer combination	FAM[2]-GACTGCGTACCAATTCACA	ATCATGAGTCCTGCTCGGTTC
9th primer combination	FAM[2]-GACTGCGTACCAATTCACT	ATCATGAGTCCTGCTCGGTGT
10th primer combination	FAM[2]-GACTGCGTACCAATTCACT	ATCATGAGTCCTGCTCGGTTC
11th primer combination	FAM[2]-GACTGCGTACCAATTCAGC	ATCATGAGTCCTGCTCGGTCG
12th primer combination	FAM[2]-GACTGCGTACCAATTCAGC	ATCATGAGTCCTGCTCGGTTA
13th primer combination	FAM[2]-GACTGCGTACCAATTCAGC	ATCATGAGTCCTGCTCGGTGC
14th primer combination	FAM[2]-GACTGCGTACCAATTCAGC	ATCATGAGTCCTGCTCGGTCC
15th primer combination	FAM[2]-GACTGCGTACCAATTCATC	ATCATGAGTCCTGCTCGGTGT
16th primer combination	FAM[2]-GACTGCGTACCAATTCATC	ATCATGAGTCCTGCTCGGTTC

EcoRI selective-amplification primers was labeled with 5'FAM.
